# Direct Evidence that Scorpion α-Toxins (Site-3) Modulate Sodium Channel Inactivation by Hindrance of Voltage-Sensor Movements

**DOI:** 10.1371/journal.pone.0077758

**Published:** 2013-11-26

**Authors:** Zhongming Ma, Jun Kong, Dalia Gordon, Michael Gurevitz, Roland G. Kallen

**Affiliations:** 1 Department of Biochemistry and Biophysics, Perelman School of Medicine University of Pennsylvania, Philadelphia, Pennsylvania, United States of America; 2 The Mahoney Institute for Neuroscience, Perelman School of Medicine University of Pennsylvania, Philadelphia, Pennsylvania, United States of America; 3 Department of Plant Molecular Biology and Ecology, George S. Wise Faculty of Life Sciences, Tel Aviv University, Ramat Aviv, Tel Aviv, Israel; Sackler Medical School, Tel Aviv University, Israel

## Abstract

The position of the voltage-sensing transmembrane segment, S4, in voltage-gated ion channels as a function of voltage remains incompletely elucidated. Site-3 toxins bind primarily to the extracellular loops connecting transmembrane helical segments S1-S2 and S3-S4 in Domain 4 (D4) and S5-S6 in Domain 1 (D1) and slow fast-inactivation of voltage-gated sodium channels. As S4 of the human skeletal muscle voltage-gated sodium channel, hNa_v_1.4, moves in response to depolarization from the resting to the inactivated state, two D4S4 reporters (R2C and R3C, Arg1451Cys and Arg1454Cys, respectively) move from internal to external positions as deduced by reactivity to internally or externally applied sulfhydryl group reagents, methane thiosulfonates (MTS). The changes in reporter reactivity, when cycling rapidly between hyperpolarized and depolarized voltages, enabled determination of the positions of the D4 voltage-sensor and of its rate of movement. Scorpion α-toxin binding impedes D4S4 segment movement during inactivation since the modification rates of R3C in hNa_v_1.4 with methanethiosulfonate (CH_3_SO_2_SCH_2_CH_2_R, where R = -N(CH_3_)_3_^+^ trimethylammonium, MTSET) and benzophenone-4-carboxamidocysteine methanethiosulfonate (BPMTS) were slowed ~10-fold in toxin-modified channels. Based upon the different size, hydrophobicity and charge of the two reagents it is unlikely that the change in reactivity is due to direct or indirect blockage of access of this site to reagent in the presence of toxin (Tx), but rather is the result of inability of this segment to move outward to the normal extent and at the normal rate in the toxin-modified channel. Measurements of availability of R3C to internally applied reagent show decreased access (slower rates of thiol reaction) providing further evidence for encumbered D4S4 movement in the presence of toxins consistent with the assignment of at least part of the toxin binding site to the region of D4S4 region of the voltage-sensor module.

## Introduction

Voltage-gated ion channels (Na_v_) are tetradomain proteins containing six (S1-S6) transmembrane segments per domain (D) with four domains that cluster around a central pore. Each domain is composed of a voltage-sensor module formed by four membrane-spanning helices (S1-S4) and a pore-forming module consisting of two membrane-spanning helices (S5-S6) with a pore-lining loop in between as revealed for sodium and potassium channels by high-resolution crystallography [1-5].

Functionally, Na_v_s are characterized by three main processes: (i) opening (activation) from a resting state when the membrane is depolarized allowing current flow; (ii) closing to the non-conducting resting state upon hyperpolarization (deactivation); and (iii) closing to a non-conducting refractory state (inactivation) in which the channel remains non-conducting in response to continuing depolarization [6]. Repolarization of the membrane is necessary for the channel to return from the inexcitable inactivated state to the resting excitable state, a process referred to as recovery from inactivation [6]. The various states represent different conformations of the channel protein that are controlled, in large part, by the highly charged S4 segments of the “voltage-sensor”, which contain 4 to 8 cationic residues in the various domains with each basic group generally separated by two hydrophobic amino acids [7,8]. While the S4 movement communicates the change in membrane potential to the remainder of the protein, the nature of the conformational alteration of the voltage-sensor remains a matter of some uncertainty.

Conformational changes of many proteins have been explored employing scanning substituted cysteine accessibility measurements (SCAM) [9-12]. A method of measuring the rate of transmembrane segment outward/inward movements has also been developed [13]. These techniques rely upon voltage-dependent changes and accessibility to side group reagents at specific cysteine reporter residues introduced by site-specific mutagenesis. The reaction at these sites with membrane-impermeable methanethiosulfonate reagents is detected by measuring electrophysiological effects such as altered channel current decay (inactivation) kinetics. The second and third basic residues of the S4 segment of Domain 4 (R2C and R3C) of the human skeletal muscle voltage-gated Na^+^ channel (hNa_v_1.4) are translocated from a cytoplasmic- to an extracellular-accessible position during depolarization based upon changes in reactivity to cytoplasmic or extracellularly administered sulfhydryl reagents [9]. The rate of reaction depends upon reagent concentration and accessibility, which is determined by the extent of exposure (position) and residence time at that position. The exposure and residence time can be altered by the duration of cyclic depolarization and hyperpolarization protocols [13].

Due to the importance of Na_v_s in excitability they are targeted by a large variety of toxins that interact at different sites, designated Sites 1 to 7 on the basis of physiological activity (e.g., alterations in conduction, activation, inactivation) and positive or negative interactions with other toxin sites [14,15]. Many of these toxins have been used as probes of channel architecture and conformation [16]. 

Scorpion α-toxins (e.g., *Leiurus quinquestriatus hebraeus* scorpion toxin LqhαIT) are members of a class of toxins (Site-3) that slow inactivation. These toxins also generally shift the voltage-dependence to more depolarizing (positive) voltages of channel availability to be activated (also referred to as h_∞_), which is an alternative reflection of channel steady-state inactivation [15,17]. Scorpion α-toxins are structurally and functionally related polypeptides (61-67 residues) containing four conserved disulfide bridges and a common βαββ conformational core [18,19]. The toxin receptor sites of channel proteins have been partly characterized by several methods including photoaffinity labeling, modification of toxin binding by site-directed reagents, monoclonal antibodies, or site-specific mutagenesis. The Site-3 toxin binding site of sodium channels includes parts of the S1-S2 and S3-S4 external linkers at D4, and S5-S6 external linker at D1 [20]. Since the stoichiometry of toxin:channel is 1:1 it is clear that these are “subsites” together comprising a single binding site with various portions of the toxins interacting with these subsites rather than being multiple (independent) binding sites [21-23]. Differential interaction with subsites is thought to account for different affinities of a given toxin for various channel isoforms. The S3-S4 external linker at D4 is dragged by the D4S4 voltage-sensor as it moves across the membrane and is preferentially involved with fast-inactivation [8,20,22,24-30]. Thus, Site-3 toxin binding might be expected to affect the D4S4 position and/or rate of movement. 

Indeed, there is evidence to support this expectation based upon decreases of gating charge and fluorescence intensity in the presence of Site-3 toxins, which suggest that bound toxin hinders S4D4 movement [20,31-33]. Gating currents are induced by depolarization and reflect the movement of charged sidechains of the channels through the membrane electric field. The gating currents decay in a double exponential time course suggesting that sodium channel voltage sensors in the various domains do not contribute equally to the gating process [27,28,34-36]. Thus, the fast component during activation is related to the movement of the voltage sensors of D1, D2 and D3 and the slow component reflects the movement of the voltage sensor of D4 [36]. In the presence of a Site-3 toxin (*Tityus serrulatus* toxin, Ts3, or *Anthopleurin A* toxin) the contribution of the slow component to the gating current decay is decreased and the total gating charge is reduced by 30%, an effect attributed to restricted movement of D4S4 arginine residues (R1, R2 and R3) [36,37]. 

Biphasic fluorescent signals (increase and then decrease) from a S3-S4 D4 loop reporter (L1439C reacted with a sulphydryl group-directed fluorophore, 5’-tetramethylrhodamine maleimide (TMRM)) are attributed to a sequential transition of the voltage sensor through two distinct environments during depolarization. In the presence of a Site-3 toxin the signal increases monophasically but the kinetics of S4 movement was not quantified. A kinetic model was proposed with two sequential open states, the first from which inactivation proceeds slowly and a second one that allows normal fast-inactivation. By preventing the full movement of the S4D4, toxin Ts3 blocks the transition to the second fast-inactivating open state, and normal inactivation is precluded. This model supports the idea that the complete movement of the S4D4 is not necessary for channel opening but is essential only for normal (fast) inactivation. In these experiments it was assumed that TMRM “tracks” the movement of D4S4 but since the reporting residue L1439C is located in the neighborhood of the toxin binding site, the effects on the fluorescence changes observed in the presence of Ts3 could be due to an effect of the toxin directly on the TMRM labeled residues themselves, rather than an effect on the movement of S4. A further caveat is that a cationic pore-blocking toxin, μ-conotoxin, interacts electrostatically with and inhibits the movement of the voltage-sensing S4 charges [38]. The cationic pore-blocker tetrodotoxin, TTX, used to enable gating current measurements is known to affect Site-3 toxin binding [30] and, thus, may confound the interpretation of the gating charge and fluorescence measurements just described [33]. Because of the possible limitations in previous experiments, we turned to SCAM to report the position of S4D4 in the absence and presence of toxin.

Since the R3 position of hNa_v_1.4 moves from an internally accessible to an externally accessible location in response to depolarization, we reasoned that if the mechanism of action of the toxin was to inhibit D4S4 translocation, the R3C reporter site might be less reactive to externally applied reagent when the membrane is depolarized in the presence of toxin [9]. We used an R3C mutant of hNa_v_1.4 to determine the D4S4 voltage-sensor position and its rates of outward and inward movements in the absence and presence of toxin LqhαIT. This toxin serves as a prototype of a subgroup of α-toxins highly active on insects and inactive on mammalian brain sodium channels but was later shown to be highly effective on the mammalian skeletal muscle sodium channel Na_v_1.4 and is, therefore, no longer considered insect-specific [39,40]. Our results support a mechanism involving hindrance of voltage-sensor movement for Site-3 toxin action and provide quantitation of the restriction of the extent of the D4S3 segment outward movement and the retardation of the kinetics of voltage-sensor movement during depolarization.

## Experimental Section

### Materials

All chemicals were of reagent grade; molecular biological reagents were obtained from New England Biolabs, Inc. (Ipswich, MA) or GIBCO-Bethesda Research Labs/Life Technologies, Inc. (Gibco-BRL-LT, Gaithersburg, MD/Carlsbad, CA). Methanethiosulfonates (MTS) were obtained from Toronto Research Chemicals, Inc. (North York, Ontario, Canada). Construction of the R3C mutant (hNa_v_1.4^R1454C^) was performed with an antisense oligonucleotide (containing a silent novel restriction site to aid screening) and a Promega kit according to the directions of the manufacturer (Promega Corp., Madison, WI). Mutant clones were identified by restriction endonuclease cleavage patterns and confirmed by nucleotide sequencing. 

### Transfection of tsA201 Cell Line

The tsA20l cell line (Sigma-Aldrich Co. LLC, St. Louis) derived from human embryonic kidney HEK 293 cells, was grown in high glucose Dulbecco's Modified Eagle's Medium (DMEM) supplemented with 10% fetal bovine-serum, 2 mM L-glutamine, penicillin (100 U/ml) and streptomycin (10 mg/mI) (Gibco-BRL-LT), in 5% CO_2_ humid atmosphere incubator. Transfection of tsA201 cells grown to 40-50% confluence on 100 mm plates was carried out using the transient calcium phosphate method with 10 μg of cDNA encoding hNa_v_1.4R3C contained in pAlter-Max (Promega Corp. Madison, WI) co-transfected with 10 μg of CD8-a, an expression plasmid for a lymphocyte surface antigen (CD8-a) [41,42]. For patch-clamp experiments, the expressing cells were used two to three days post-transfection and identified by their decoration with anti-CD8-coated beads (Dynabeads M450 CD8, Dynal A.S., Oslo, Norway). Approximately 50% of the cells expressed large Na currents (>500 pA at −10 mV) in typical experiments. 

### Electrophysiology Patch-clamp Method

Macroscopic sodium currents from transfected cells were recorded using the whole-cell patch-clamp technique [43]. Patch electrodes were made from 8161 Corning glass coated with Sylgard (Dow-Corning) to minimize capacitance. A good voltage clamp was accomplished using low resistance-electrodes (<2 mΩ) and a series resistance compensation was performed to values >80% to minimize voltage-clamp errors (<3 mV) with an Axopatch 200B patch-clamp amplifier (Axon Instruments/Molecular Devices, LLC, Sunnyvale, CA). Sodium currents were corrected by leak subtraction: typically, the steady-state passive membrane response to a voltage step is subtracted from the output. Voltage-clamp command pulses were generated by microcomputer using pCLAMP software v 6.0 (Axon Instruments/Molecular Devices). Recorded membrane currents were filtered at 5 kHz, sampled at 25 kHz. For whole-cell recording, the patch pipette contained (mM): 35 NaCl; 105 CsF; 10 EGTA; 10 Cs-HEPES (pH 7.4). The bath solution contained (mM): 150 NaCl; 2 KCl; 1.5 CaCl_2_; 1 MgCl_2_; 10 glucose; 10 Na-HEPES (pH 7.4). Experiments, unless noted otherwise, were performed at room temperature (20-22 ^O^C) and at a holding potential of −120 mV ten min after breaking the membrane. To distinguish residues exposed only when a channel is open, and not when inactivated, or *vice versa*, solution application was switched on a submillisecond timescale between two converging solution inputs applied to an excised patch. 

### Measurements of Channel Modification by Toxin or MTS

The toxin was stored at 100 mM in aliquots at −20° C and incubated with tsA201 cells for 30 min prior to formation of giga-Ω seals. After establishing continuity between the cytoplasm and micropipette solution, current measurements were initiated. Toxin-containing or toxin-free solutions were perfused around the cell from a ~20 μm diameter micropipette with the solution velocity controlled by air pressure and gravity. Channels exhibiting slowed inactivation and increased residual currents compared with controls are those with toxin bound or cysteine-modified (by MTS reagent), both of which are all-or-none for each channel. The time course of channel modification by toxin or MTS showed a progressive increase in the slower of two exponential fits to the current inactivation curves. During modification an increasing fraction of channels is being modified (*F*_*mod*_), beginning at zero and ending at one. The biphasic current decays of toxin- or MTS-modified channels were fit to a double exponential equation, I=FF(e((t−to)/τF))+FS(e((t−to)/τS))+Ires where *I* and *I*_*res*_ are the current and current offset, respectively, *F*_*F*_ *, F*_*S*_, τ_*F*_ and τ_*S*_ are the amplitudes (weighting factors) and time constants of the fast and slow exponentials, respectively, *t*_*o*_ is the time offset and the *F*_*mod*_ = *(F*_*S*_)/*(F*_*S*_ + *F*_*F*_). The slow component of current decay increases with time and the exponential time dependence of the increase in the fraction of toxin- or MTS-modified hNa_v_1.4s, (*F*_*S*_)*/*(*F*_*S*_ *+ F*_*F*_), yields the time constant for channel modification by toxin or MTS, τ_*Tx*_ or τ_*MTS*_, the inverses of the rate constants *ρ*_*Tx*_ or *ρ*_*MTS*_. We have shown that the relative contributions of the two exponentials in the presence of intermediate concentrations of LqhαIT or during the time course of MTS modification are independent of current amplitude by decreasing the latter with increasing concentrations of TTX, a channel blocker in separate experiments (data not shown). Whole-cell data were analyzed by a combination of pCLAMP and Excel programs.

### Analysis of Data

**Figure 1:**
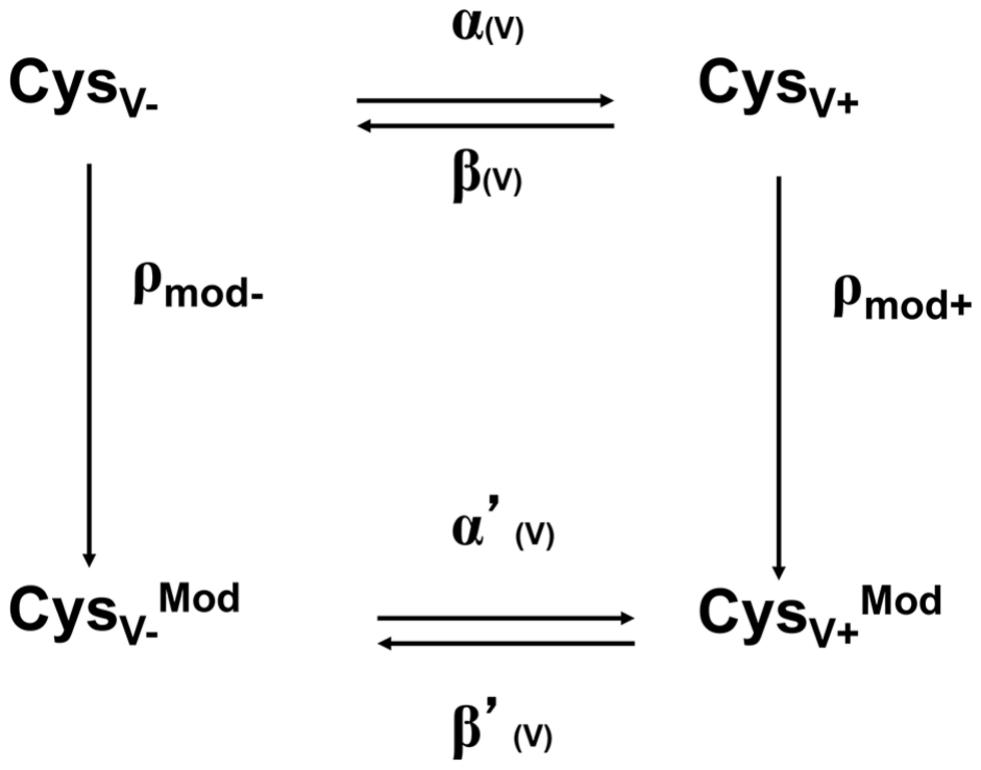
Voltage-dependent modification of Na_v_1.4 by MTS reagent. The chemical modification reactions for the two states (inside at negative and outside at positive voltages) are designated, ρ_mod_- and ρ_mod_+ and lead to two corresponding modified cysteine states, Cys_V-_^Mod^ and Cys_V+_^Mod^. The rates of movements of the voltage sensor are denoted by voltage dependent rate constants α(V) and β(V) for unmodified channels and α’(V) and β’(V) for modified channels.

The observed R3C modification rate, *ρ*_*mod*_, for applied reagent depends upon the concentration of MTS reagent, the extent of exposure of Cys^1454^ (R3C), and the duration of exposure to the MTS reagent: the latter two are determined by the voltage and the cycling times between hyperpolarized and depolarized states (Figure 1). For the simplest case, Cys^1454^ has two accessibilities: one at hyperpolarized (negative) or the other at depolarized (positive) voltage extremes, designated Cys_V-_ and Cys_V+_ (Figure 1, upper row). 

The chemical modification reactions for the two states (inside at negative and outside at positive voltages) are designated, ρ_mod-_ and ρ_mod+_ and lead to two corresponding irreversibly modified cysteine states, Cys_V-_^Mod^ and Cys_V+_^Mod^ (Figure 1, lower row) [13]. The voltage-dependent rates of movement of the voltage sensor (accessibility) are denoted by voltage-dependent rate constants α(V) and β(V) for unmodified channels and α’(V) and β’(V) for modified channels. When *ρ*_*mod-*_ and *ρ*_*mod+*_ are known from experiments at hyperpolarization and depolarization extremes, it is possible to estimate the external reagent accessibility of R3C from the apparent rate of modification at different membrane potentials. In addition, by cycling between two voltages with different exposures the rate of movement of the voltage-sensor can be determined. The reason it is possible to control the observed modification rates by altering the reagent concentration is because for a simple bimolecular reaction between the thiolate and the reagent, *ρ*_*mod+*_ and *ρ*_*mod-*_ are each linear functions of reagent concentration. If modification is rate-limiting for a fixed reagent concentration with *ρ*_*mod+*_ and *ρ*_*mod-* <<_ α(V) and β(V), the time course of modification at any voltage will be pseudo-first order with an overall modification rate equal to a weighted sum of *ρ*_*mod+*_ and *ρ*_*mod-*_ in which the weighting factor for each rate is the steady-state probability (Pr) of the channel being in either of its two conformations, Cys_V+_ or Cys_V-_, that is, *ρ*_*mod*_ = [Pr^CysV+^ x *ρ*_*mod+*_] + [Pr^CysV-^ x *ρ*_*mod-*_]. By using large depolarizations or hyperpolanzations all channels can be put into either the Cys_V+_ or the Cys_V­_ state and either *ρ*_*mod+*_ or *ρ*_*mod-*_ can then be determined directly. When these two rates are known, the overall rate of modification (*ρ*_*mod*_) at different membrane potentials can be used to infer the voltage-dependent accessibility (exposure probability, *p*) of a specific cysteine residue. The steady-state exposure probabilities of R3C at V_1_ and V_2_ are *p*_*∞,1*_ and *p*_*∞,2*_, which can be estimated from graphs of *ρ*_*mod*_ vs. voltage from a Boltzmann fit. The cysteine exposure and burial rates are assumed to be exponential at the depolarized and hyperpolarized voltages (Eqs. 1 and 2) with rate constants for transitions from accessibility to inaccessibility *ρ*_*1*_ and from inaccessibility to accessibility, *ρ*_*2*_, at depolarized (V_1_) and hyperpolarized voltages (V_2_) [13]. The time course of exposure probability at each voltage is exponential according to:

$$P_{V1}(t)=p_{\infty ,1}+(p_{init,1}-p_{\infty ,1})exp(-\rho_{1}t)$$ (1)

$$P_{V2}(t)=p_{\infty ,2}+(p_{init,2}-p_{\infty ,2})exp(-\rho_{2}t)$$ (2)

The rates *ρ*_*1*_ and *ρ*_*2*_ are the inverses of the time constants for changes in cysteine accessibility at V_1_ and V_2_ depicting the rates of exposure and burial, respectively, of the cysteine residue. The initial conditions of exposure probability at the moment of changing the voltage are *p*_*init,1*_ and *p*_*init,2*_

$$P_{init,2}\text{ = }\frac{p_{\infty ,1}+(p_{\infty ,2}-p_{\infty ,1})exp(-\rho_{1}\Delta t)-p_{\infty ,2}(-exp(-(\rho_{1}+\rho_{2})\Delta t)}{1-exp(-(\rho_{1}+\rho_{2})\Delta t)}$$ (3)

$$P_{init,1}=p_{\infty ,2}+(p_{init,2}-p_{\infty ,2})exp(-\rho_{2}\Delta t)$$ (4)

for depolarizing and hyperpolarizing pulses, respectively, and depend on pulse duration (evaluated with Eqs. 3 and 4 for a pulse duration *Δt*, which is applicable because the conformational changes of the voltage-sensor do not occur instantaneously upon the change in voltage). The values of *p*_*init,1*_ and *p*_*init,2*_ for a pulse duration *Δt* can be substituted into Eqs. 1 and 2, which can then be integrated to calculate the exposure probability for a pulse train of arbitrary *Δt*. The integral for one cycle of depolarization and hyperpolarization is given by equation 5. 

$$\int_{0}^{\Delta t}\left[P_{v1}(t)+P_{v2}(t)\right]dt=(p_{\infty ,1}+p_{\infty ,2})\Delta t$$

$$+\left[p_{init,1}-p_{\infty ,1})(1-exp(-\rho_{1}\Delta t\right]/\rho_{1}$$

$$+\left[p_{init,2}-p_{\infty ,2})(1-exp(-\rho_{2}\Delta t\right]/\rho_{2}$$ (5)

This integral is normalized by dividing it by *Δt*, which allows the direct comparison of exposure probability as a function of *Δt* and can be used to estimate the exposure (reagent-accessible) and burial (reagent-inaccessible), rates, *ρ*_*1*_ and *ρ*_*2*_ (i.e., rate of movements of D4S4 voltage-sensor), from the effect of *Δt* on *ρ*_*mod*_ for externally applied reagent (see Results section). At progressively smaller *Δt* values the depolarizing duration does not allow the cysteine position to attain the exposed position so the reaction rate will gradually tend toward that of the inaccessible state [13]. 

## Results

### Channel Inactivation is Slowed by Covalent Modification of R3C

**Figure 2:**
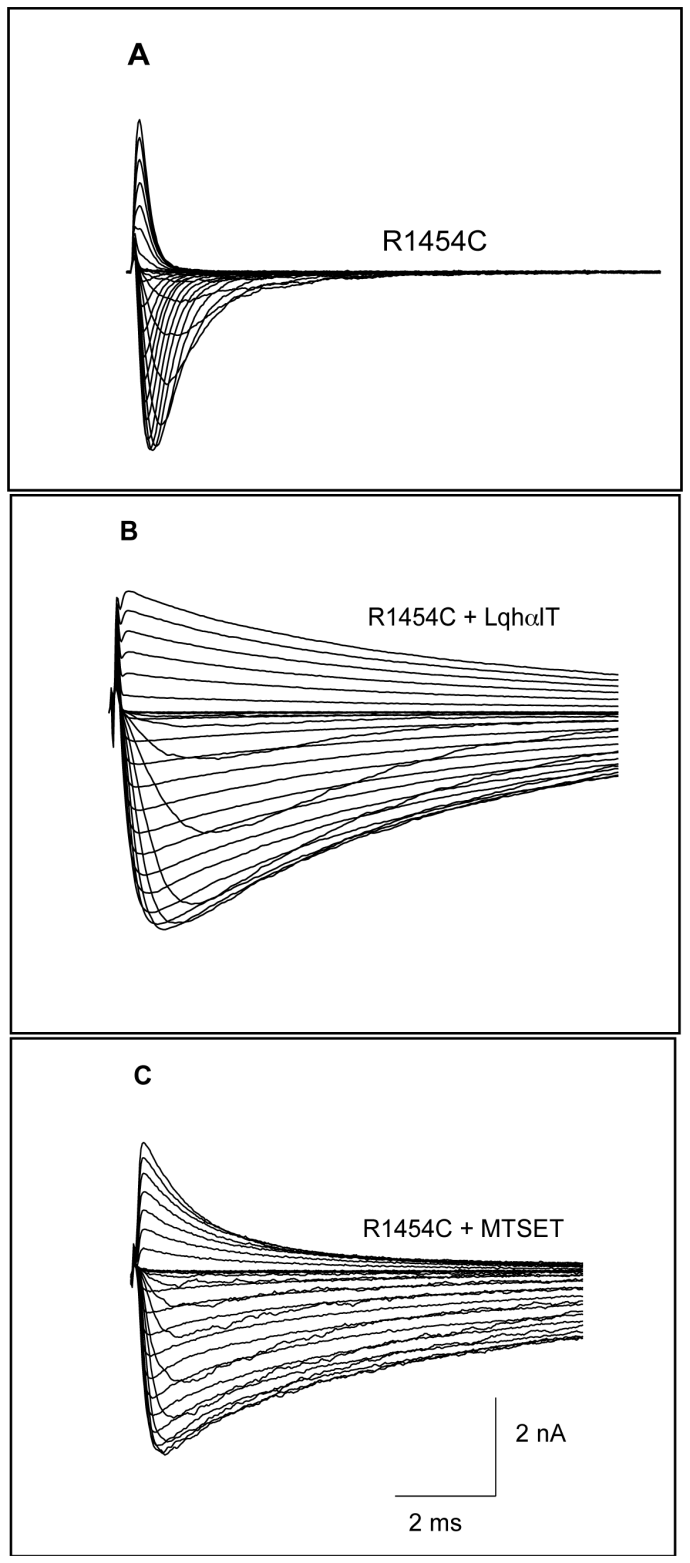
Whole-cell sodium currents of slowed hNa_v_1.4 inactivation in presence of toxin or following MTS modification. The channels are activated by 40 ms depolarization in 5 mV increments from −90 to +65 mV. (**A**) R3C; (**B**) R3C with 100 nM LqhαIT in bath solution incubated at room temperature for 30 min prior to patching the cell. (**C**) R3C after 10 min treatment with 20 µM extracellular MTSET (cysteine side-chain modification is complete). HP −120 mV.

Electrophilic MTS reagents are attacked by the cysteine thiolate anion to form a mixed disulfide with the addition of a side-chain determined by the nature of the reagent (cationic for methanethiolsulfonylethyltrimethylammonium, MTSET) [44]. The modification of R3C (hNa_v_1.4^R1454C^) by a thiol reagent slows the kinetics of inactivation for the fraction of channels, *F*_*S*_, that are modified as the reaction progresses. Normalized *F*_*S*_ values as a function of time progress from zero to unity monoexponentially providing the rate of modification. This is illustrated by the reaction of R3C with MTSET (20 μM), which slows the decay of current simultaneously with the appearance of a pedestal or residual current (Figure 2C), similar to the action of scorpion α-toxins such as LqhαIT (Figure 2B) when compared with unmodified R1454C (Figure 2A). 

### Kinetics of Modification from Externally Applied MTS in the Absence of Toxin

**Figure 3:**
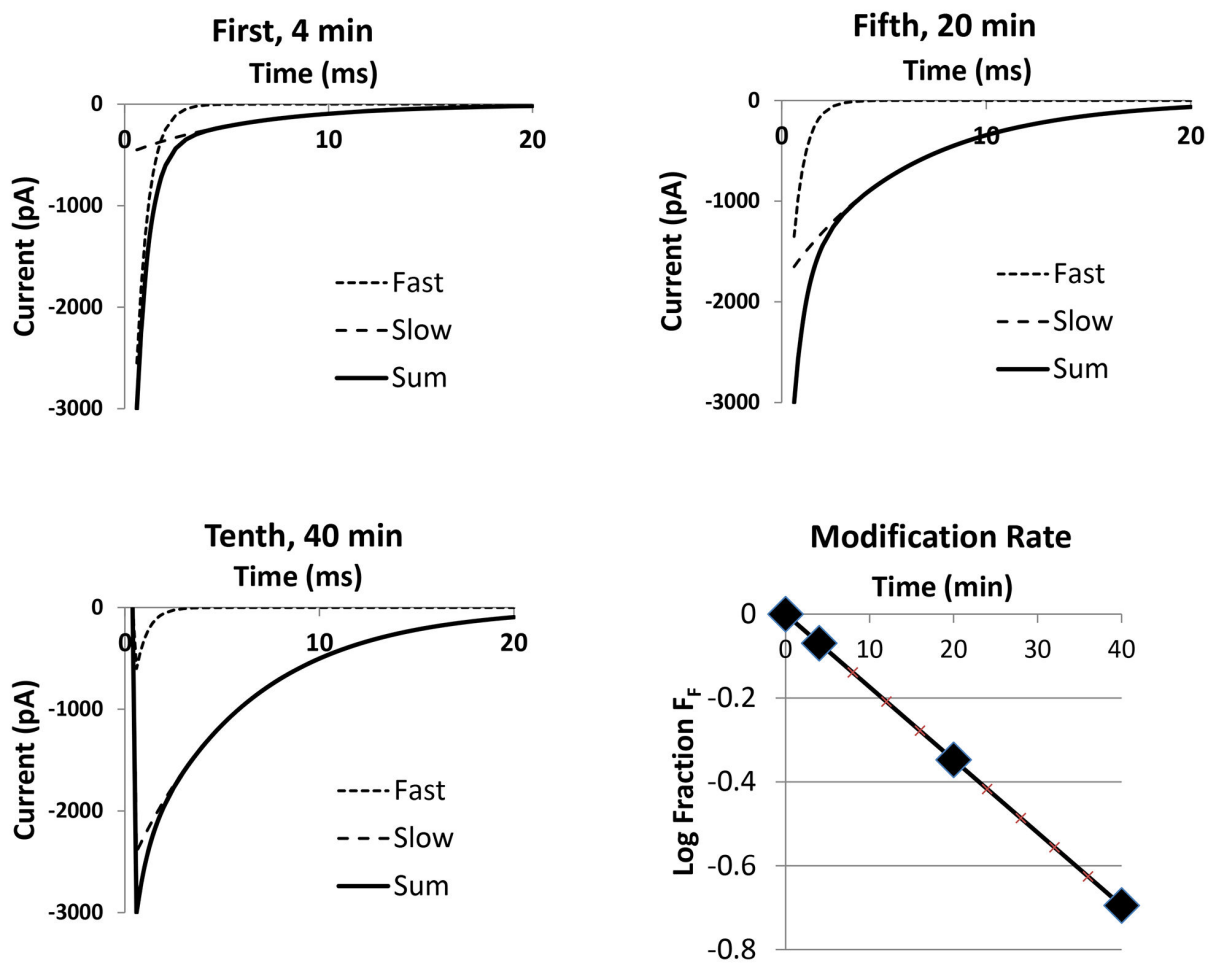
Time course of MTSET modification in the absence of toxin at −120 mV. The current decay kinetics contains fast and slow exponential components, representing unmodified and modified channels, respectively. The time dependence of the decrease in the fraction of total current contributed by the fast component, *F*_*F*_, provides the time constant for modification, τ_MTS_. At the three times shown: *F*_*F*_ = 0.85, 0.45 and 0.20. τ_F_ = 0.6 s; τ_S_ = 6 s; τ_MTS_ = 25 s; [MTSET] = 20 μM. HP −120 mV. Test potential −20 mV.

Unobstructed thiolate anions with pK_a_ values in the range of those of cysteine side-chains in proteins react in fractions of a second with MTS reagents at the usual concentrations (20-50 μM) employed at physiological pH [45]. In contrast, cysteine thiolate anions that react at slower rates are partially inaccessible. The biphasic current decays in the presence of toxin can be fit to a double exponential (Experimental Section) enabling measurement of the change in accessibility by the alteration of cysteine modification rate (Figure 3). The observed value of the time constant of 25 min for cysteine modification is ~170-fold slower than that of unobstructed thiolate anions because the R3C site is less accessible at a membrane potential of –120 mV (assuming no pK_a_ value or other changes). A similar set of biphasic kinetics with increasing *F*_*S*_ as a function of time is seen after the addition of toxin reflecting the course of toxin binding: the rate constant for binary complex formation can be obtained from the time dependence of *F*_*S*_: k_on_ ~ 5.3 x 10^5^ M^-1^s^-1^, t_0.5_ ~ 2-20 s at –120 mV and [Tx] = 50-500 nM (data not shown). Therefore, toxin binding is much faster than MTS modification rate at the R3C site and does not interfere with the MTS kinetic measurements.

### Kinetics of Channel Modification by MTS Reagent in the Presence of Externally Applied Toxin

**Figure 4:**
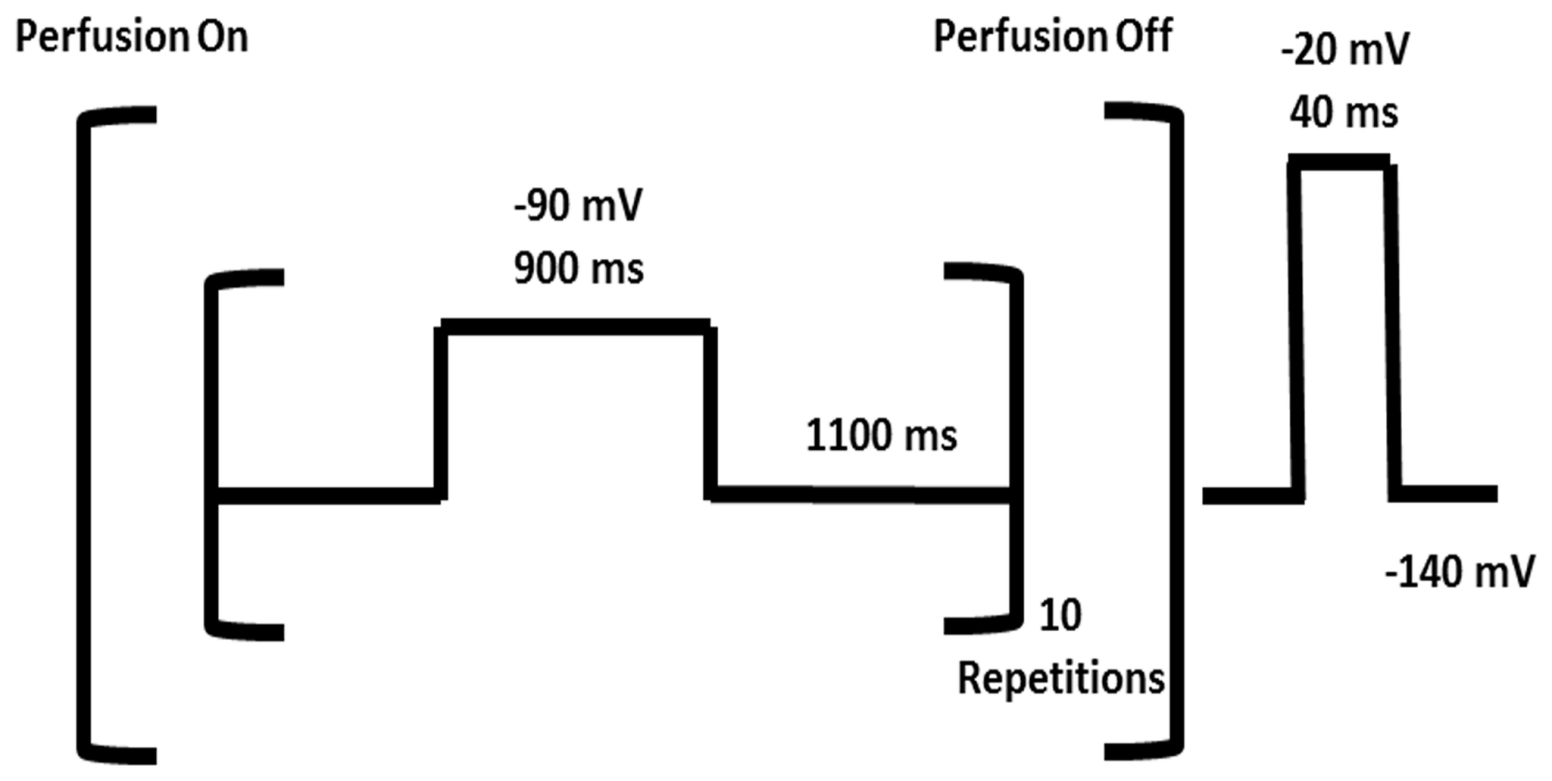
Protocol for voltage-dependent modification of hNa_v_1.4. The experiment involved ten oscillations between two voltages: 900 ms at the depolarizing test voltage (voltage sensors outward, fast-inactivated state) and 1100 ms at the hyperpolarizing holding potential (HP) (voltage sensors inward, recovered from inactivation). During these oscillations the channel was perfused with toxin/MTS solution. The sampling of the currents took place during perfusion with control solution after ten of the previously described oscillations (every 20 s) during a 40 ms depolarization to –20 mV allowing the channel to open and inactivate.

**Figure 5:**
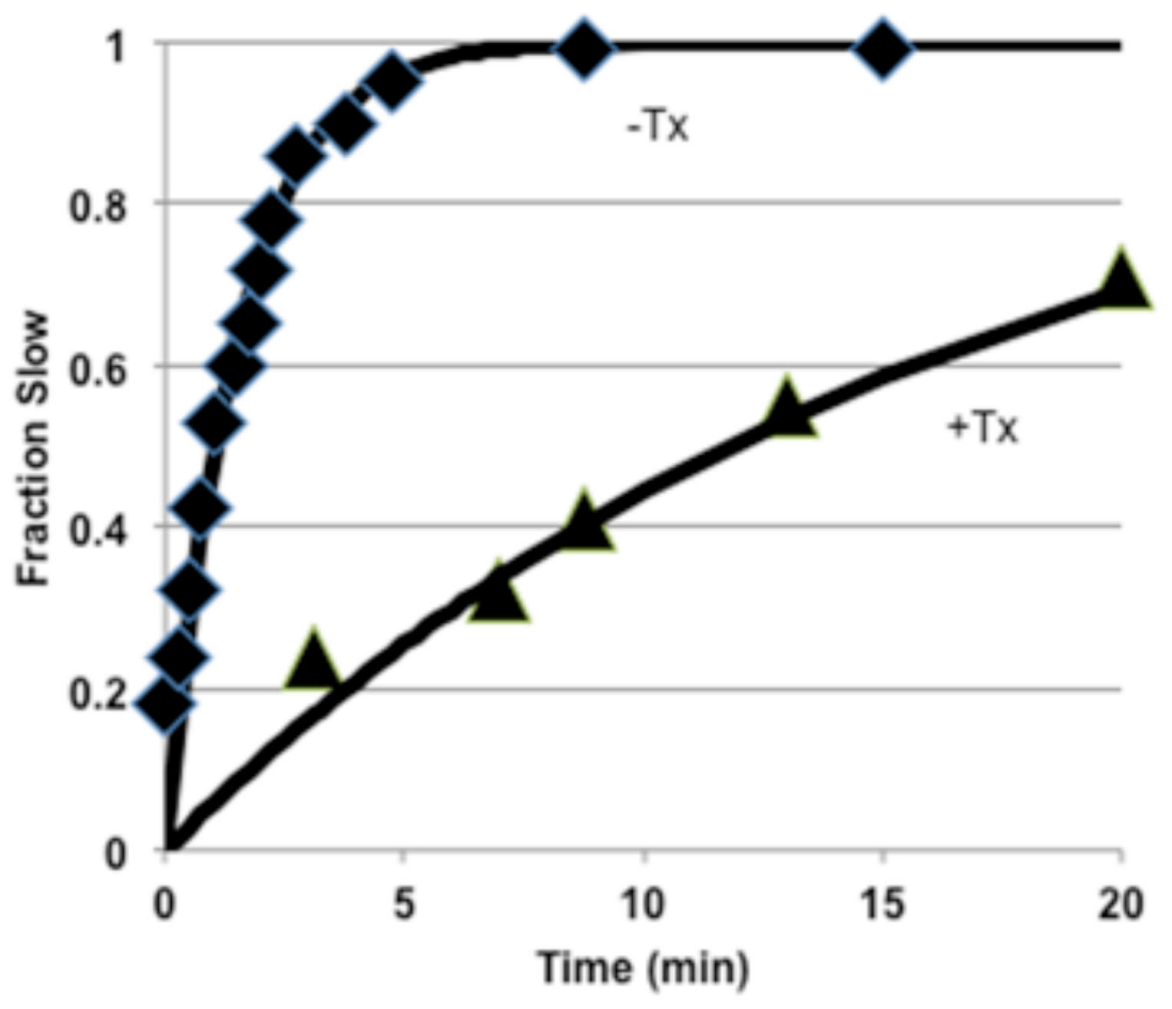
Kinetics of R3C modification with externally applied MTS in absence and presence of toxin. The solid lines are calculated with the time constants (◆) LqhαIT absent, τ_MTS_ = 1.5 min; (▲) LqhαIT present (50 nM), τ_MTS_ = 17 min and F_S_ = 1-exp(-t/τ_MTS_). Holding potential = –140 mV, test potential –90 mV. The protocol is described in the text (Figure 4). [MTSET] = 20 μM.

**Figure 6:**
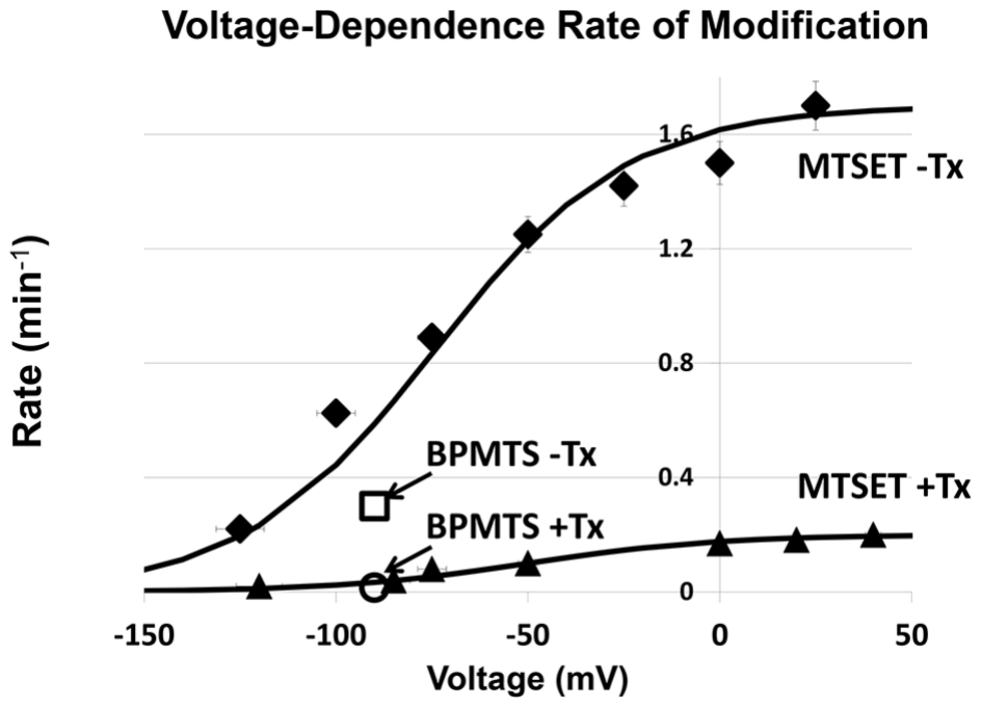
Voltage-dependence of kinetics of hNa_v_1.4R3C modification, ρ_mod_, with externally applied MTSET (20 μM). (◆) Toxin absent; (▲) toxin present [LqhαIT] = 500 nM. The solid lines are calculated with Rate = ρ_mod_^Max^/(1+exp(-(V-V_0_._5_/25) and V_0.5_ = −74 mV (**−**Tx) and +50 mV (+Tx); ρ_mod_^Max^ = 1.7 min^-1^ (**−**Tx) and 0.2 min^-1^ (+Tx) (Table 1). [BPMTS] = 500 nM −90 mV, (**☐**) –Tx and (○) +Tx. Error bars are generally hidden by the symbols.

Since both toxin and MTS reactions slow current decay, the protocol employed required the removal of toxin during the analysis of the extent of R3C modification by MTS. For reaction with MTS from the extracellular compartment this was accomplished by controlling the rates of side-chain reaction by adjusting the MTS concentration (pseudo-first order reaction) so that toxin binding and unbinding are always rapid relative to the kinetics of the MTS reaction. The experiment involved ten oscillations between two voltages: 900 ms at the depolarizing test voltage (voltage sensors outward) and 1100 ms at the hyperpolarizing holding potential (HP) (voltage sensors inward). The depolarization allows channels to open and enter mostly into fast-inactivated states, while the subsequent hyperpolarization allows channels to recover from inactivation. During these oscillations the channels were perfused with toxin/MTS solution. The sampling of the currents took place during perfusion with control solution after 10 of the previously described oscillations (every 20 sec) during a 40 msec depolarization to –20 mV allowing channels to open and inactivate (Figure 4). With virtually saturating toxin concentration (50 nM, K_d_^LqhαIT^ ~ 4 nM) at one representative voltage (–90 mV), externally applied MTSET (20 μM) reacted ~8-fold more slowly in the presence of LqhαIT (*τ*_*MTS*_^-Tx^ = 1.5 min and *τ*_*MTS*_^+Tx^ = 12 min, Figure 5). Note that slowing of modification rate at –90 mV was also observed for BPMTS (Figure 6, (**☐**) –Tx and (○) +Tx), which adds an anionic bulky aromatic side-chain to R3C (Figure 6). The steady-state exposure probabilities of R3C, p_∞,1_ and p_∞,2,_ are obtained from the plateau values of the voltage-dependence of the modification rates, which follow a Boltzmann relationship consistent with two states, Cys_V-_ (inaccessible) and Cys_V+_ (accessible), with markedly different extracellular MTS reactivities. For example, the rates of modification at −125 and −25 mV are 0.22 and 1.42 min^-1^ at 20 μM MTSET, respectively, reflecting these differences in accessibility in the absence of toxin.

Experiments with internally applied MTSET also demonstrated rates of modification of R3C that were many-fold slower in the presence of toxin but the kinetics were sigmoid suggesting a complexity that was not further pursued (data not shown).

### Voltage-Dependence of Channel Modification by MTS Reagent in the Absence and Presence of Externally-Applied Toxin

Analysis of the voltage-dependence of the MTS reaction using a similar protocol as that shown in Figure 5 at different voltages revealed that the toxin slowed the MTSET reaction at all voltages studied (Figure 6). During these experiments we have compensated for the fact that the toxin:channel binary complex dissociation constant, K_d_, is voltage-dependent varying from 5 to 150 nM over the range –120 to +50 mV by adjusting the concentration of toxin to be ~10K_d_ to maintain >90% of the channel with toxin bound [46]. 

### Kinetics of Voltage-Sensor Movements in Absence and Presence of Toxin

**Figure 7:**
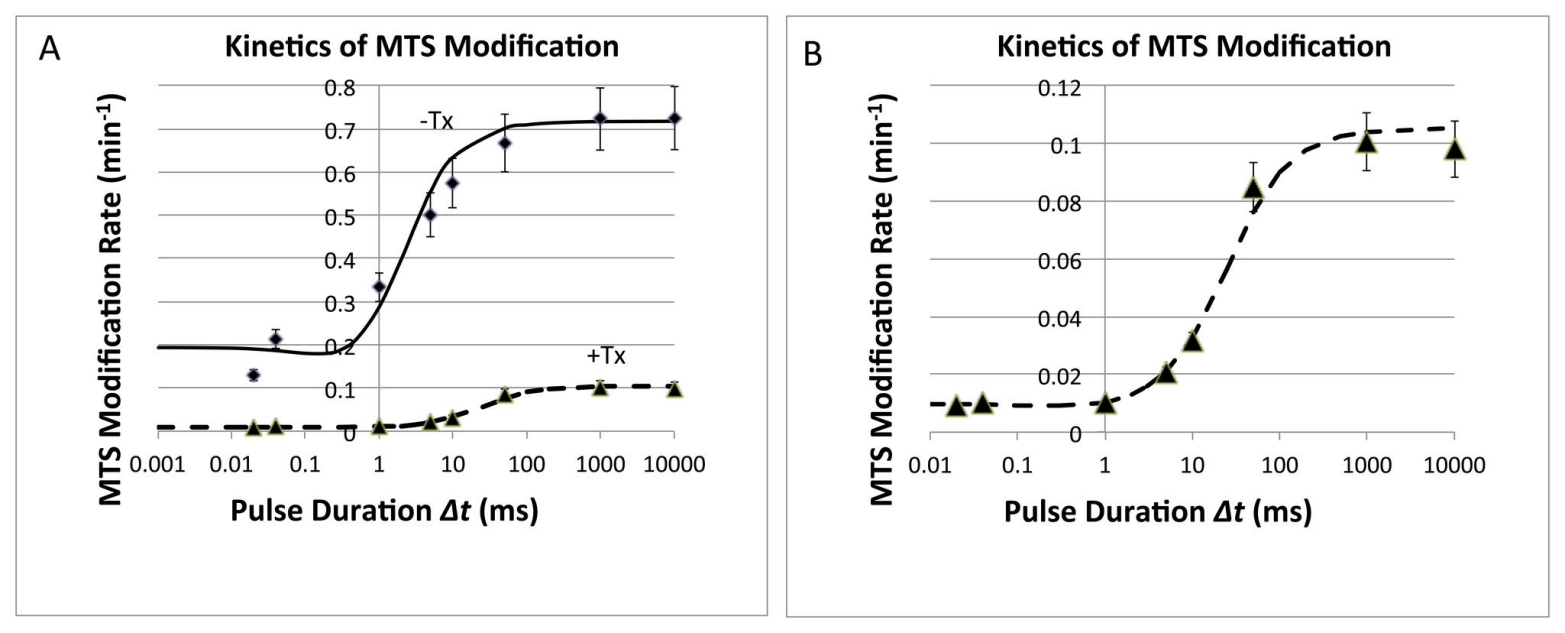
Modification rate (externally applied MTSET, 20 μM) vs. durations of individual depolarizations from -140 to -40 mV The solid lines are the best fit to the equations 1-5 as described in the text with parameters from Table 1.

The pulse durations (*Δt*) were systematically varied to estimate the voltage-dependent rate constants for R3C movement at depolarized (-50 mV, 900 ms) and hyperpolarized (-140 mV, 1100 ms) voltages. The data for this experiment show *ρ*_*mod*_ as a function of individual pulse duration (*Δt*). When pulses were sufficiently long, *ρ*_*mod*_ was independent of pulse duration over a range of several orders of magnitude (~500 to 10,000 ms). However, when individual pulse durations approached the kinetics of cysteine exposure (Figure 7), *ρ*_*mod*_ decreased, because this cysteine residue did not have enough time to get fully exposed during short depolarizations. Under such conditions the kinetics of outward and/or inward voltage-sensor movement could be measured by the variation in rate constants for MTS reaction vs. *Δt* providing estimates of the rate of voltage-sensor movement from buried to exposed and *vice versa* where *ρ*_*1*_ and *ρ*_*2*_ are the rate constants for changes in cysteine accessibility at V_1_ and V_2_, respectively (see Experimental Section) [13]. The solid curve plots the normalized integral of Eq. 5, using the best-fit time constants for exposure and burial of the cysteine (Table 1). The tendency to level off at hyperpolarized voltages rather than have a value of zero indicates that the burial was not infinitely fast at -140 mV for R3C. The accessibility can be fit reasonably well by a Boltzmann function (solid curve), consistent with the simple two-state gating model (Figure 1) [13]. The values in the absence of toxin compare well with earlier studies [13].

**Table 1:** Parameters for Calculated Curves (Equation 5).

**Parameter**	**No Toxin**	**Toxin**	**Ratio**
V_0.5 (mV)_	-74	-50	
ρ_mod_^app^ (min^-1^)	1.7	0.2	8.5
	***Δt***		
P_∞,1_	0.71	0.103	6.9
P_∞,2_	0.004	0.002	1.9
ρ_1_ (ms^-1^)	0.723	0.065	11.2
ρ_2_ (ms^-1^)	4.9	2.25	2.2

Voltage from Figure 6; *Δt* from Figure 7.

## Discussion

### R3C (R1454C) Reactivity To Externally Applied MTS Is a Reporter of D4S4 Position and Movement

Reactivity of site-specifically introduced cysteine residues has been a powerful tool to probe the conformation of a protein, the environment around that substitution site and changes in either of them (SCAM) [12,44,47]. In the case of voltage-sensitive sodium channels, the formation of a disulfide bond at R3C upon reaction with MTS reagents is readily detectable by a marked slowing of current decay kinetics (Figure 2). Using the MTS probe, the cysteine position at R3 was shown to change from an interior to an exterior accessible location with depolarization based upon differences in reactivity to MTS thiol reagents when presented to the channel from either the extracellular or cytoplasmic sides [9,10]. The rate constants for unobstructed thiolate anion modification appear to be voltage-independent, with values ~10^6^ M^-1^s^-1^, for pK_a_ values of 8 - 9.5 for cysteine sidechains of typical proteins, consistent with observed voltage-dependent kinetics being due not to different chemistries but rather to differences in accessibility to the MTS reagent [45]. This conclusion depends upon the assumption that the apparent rate constants, *ρ*_*mod*_, are directly proportional to accessibility. For the R3C site of the hNa_v_1.4 channel the *ρ*_*mod*_ values are smaller by factors of 20- to 40-fold in the absence of toxin compared with those of unobstructed thiolate anions indicating greater protection of this site within the context of the channel protein at –40 mV. The values of *ρ*_*mod*_ approach minimum values at hyperpolarized voltages supporting the view that the R3C cysteine becomes less accessible to extracellular MTSET at progressively hyperpolarized voltages. 

### R3C (R1454C) Reactivity To Externally Applied MTS Is Diminished in the Presence of Toxin

Toxin-modified channels exhibit shifts in the midpoint of the availability curve (h_∞_) of ~25 mV in the depolarizing direction (with <1 e_o_ difference in slope factors data not shown), which is similar to the difference in midpoints between the curves for voltage-dependence of the rates of MTS modification in the absence or presence of toxin. Since G-V curves and activation kinetics are not significantly affected by the toxin, we conclude that D4S4 segment transitions largely involving inactivation are those affected by the toxin [8,26,27,33,48]. Measurements in the range –140 mV to +40 mV reveal that the values of *ρ*_*mod*_ continue to be consistently smaller by a factor of about ten-fold in the presence of toxin (Figure 6), consistent with greater limitation of R3C site accessibility in the presence of toxin when hNa_v_1.4 is in both hyperpolarized conformations and the depolarized states in the voltage range studied. The fact that reaction rates of two very different MTS reagents in terms of size, charge, and hydrophobicity (MTSET and BPMTS) are each slowed about ten-fold at –90 mV in the presence of toxin suggests that access to the R3C site is not blocked by LqhαIT but, rather, that the R3C site is less available when the toxin is bound to the channel, that is, the voltage-sensor is limited with regard to its ability to progress outward upon depolarization (i.e., even at the most positive voltages studied the maximal excursion of the R3C site is at a more interior, less reactive position than in the absence of toxin). Thus, voltage-sensor mobility is hindered by the toxin.

### Rates of D4 Voltage-Sensor Movement in the Absence and Presence of Toxin

When MTS reactions are carried out during cyclical depolarizations (−40 mV) and hyperpolarizations (−140 mV) with varying cycle times (*Δt*) the rate of voltage-sensor movement can be measured. When the duration of individual depolarizations is much longer than the time required for cysteine accessibility (R3C) to reach steady state, the rate of modification by a fixed concentration of cysteine reagent does not depend on the duration of individual pulses. This is because the total exposure time (i.e., the integral of the probability of cysteine exposure) will be independent of pulse duration when ρ_mod+_ and ρ_mod- <<_ α(V) and β(V) (Figure 1). However, when the pulse duration approaches the voltage-dependent kinetics of R3C exposure, the rates of channel modification become dependent upon the pulse duration due to the fact that for the shorter depolarizations, the cysteine residue has less probability of being exposed. These data may be used to estimate the time constants of cysteine exposure and burial at the two voltages used for pulsing, since the transition between less accessible and more accessible states will have exponential kinetics after a step of voltage. The integral of exposure probability (i.e., accessibility) can be determined analytically if the time constants of exposure and burial are known from the effects of pulse duration on *ρ*_*mod*_, because *ρ*_*mod*_ is directly proportional to accessibility. 

In measuring the kinetics of the change in R3C accessibility (by the pulse-duration method based on the exposure probability as a function of *Δt*) we found that the rate constant for S4 outward movement (*ρ*_*1*_) was slowed ~10-fold consistent with the conclusion that toxin hinders the outward voltage-sensor movements (Figures 5 and 6) [13,33]. Inward voltage-sensor movements were also slowed but less so (Table 1).

In conclusion, the R3C site of D4S4 is less accessible from outside in the presence of toxin indicating that the voltage-sensor movement is hindered from attaining its maximum outward excursion even at very positive voltages and there is a 10-fold slower rate of outward movement revealed by the pulse-duration method in the presence of toxin. 
